# Robotic-Assisted Portal Vein Resection and Reconstruction in Hepato-Pancreato-Biliary Surgery with a Video

**DOI:** 10.1245/s10434-025-18145-4

**Published:** 2025-09-01

**Authors:** Perrine Côme, Farouk Mourthadhoi, Bertrand Le Roy

**Affiliations:** 1 Service de chirurgie digestive et oncologique, CHU Saint-Etienne, Saint-Étienne, France; 2 Centre pour l’lnnovation en Cancérologie de la région Lyonnaise (CICLY), EA UCBL/HCL 3738, Claude Bernard University Lyon 1, Lyon, France

Pancreatic adenocarcinoma (PA), accounting for 90% of pancreatic tumors, is projected to become the second leading cause of cancer-related death in Europe and the USA by 2030.^1^ Although biliary tract cancers remain less frequent, their incidence is also rising. These alarming trends have intensified the demand for effective surgical strategies that ensure oncologic rigor while minimizing morbidity. Minimally invasive approaches—particularly robotic surgery—are increasingly adopted in hepatopancreatobiliary (HPB) oncology. High-volume series and meta-analyses have demonstrated that robotic pancreatic or hepatic resections achieve comparable R0 resection rates and survival outcomes to open surgery, with reduced perioperative morbidity.^2–4^ Furthermore, recent reports have demonstrated the feasibility and safety of complex venous resections and reconstructions during robotic pancreatoduodenectomy—whether using bovine pericardial grafts for extensive portomesenteric involvement or performing primary venous anastomoses without graft in selected cases of superior mesenteric vein (SMV) invasion.^5–7^

This video illustrates three robotic techniques for venous resection during major HPB surgery:

1. Lateral venous resection with primary closure
2. Resection with bovine pericardial patch reconstruction
3. End-to-end porto-portal anastomosis

Each technique is performed in accordance with oncologic principles, with emphasis on vascular control, precise dissection, and reconstruction using 5-0 Prolene or GoreTex sutures. Strategies, such as temporary unclamping and intraoperative Doppler, are used to confirm patency before completing reconstruction. Clamping times ranged from 30 to 70 min, and postoperative imaging confirmed adequate portal vein flow and caliber in all cases.

This demonstration confirms the feasibility and safety of robotic venous resections in HPB oncology, reinforcing its role as a technically sound and oncologically acceptable alternative to open surgery.

## Supplementary Information

Below is the link to the electronic supplementary material.

Supplementary file1 (MP4 156626 KB)
